# Is There More Than One Road to Nevus-Associated Melanoma?

**DOI:** 10.5826/dpc.1002a28

**Published:** 2020-04-03

**Authors:** Roberta Vezzoni, Claudio Conforti, Silvia Vichi, Roberta Giuffrida, Chiara Retrosi, Giovanni Magaton-Rizzi, Nicola Di Meo, Maria Antonietta Pizzichetta, Iris Zalaudek

**Affiliations:** 1 Dermatology Clinic, Hospital Maggiore, University of Trieste, Italy; 2 Department of Clinical and Experimental Medicine, Section of Dermatology, University of Messina, Messina, Italy; 3 Division of Oncology B, CRO Aviano National Cancer Institute, Aviano, Italy

**Keywords:** nevus-associated melanoma, melanoma, melanogenesis, dermoscopy, eosinophilia

## Abstract

The association of melanoma with a preexisting nevus is still a debated subject. Histopathological data support an associated nevus in approximately 30% of all excised melanomas. The annual risk of an individual melanocytic nevus becoming malignant is extremely low and has been estimated to be approximately 0.0005% (or less than 1 in 200,000) before the age of 40 years, to 0.003% (1 in 33,000) in patients older than 60 years. Current understanding, based on the noticeable, small, truly congenital nevi and nevi acquired early in life, is that the first develops before puberty, presents with a dermoscopic globular pattern, and persists for the lifetime, becoming later a dermal nevus in the adult. In contrast, acquired melanocytic nevi develop mostly at puberty and usually undergo spontaneous involution after the fifth decade of life. The purpose of this review is to analyze the data of the literature and to propose, on the basis of epidemiological and clinical-dermoscopic characteristics, a new model of melanogenesis of nevus-associated melanoma.

## Introduction

The association of melanoma with a preexisting nevus is still a debated subject. Histopathological data support an associated nevus in approximately 30% of all excised melanomas [1]. It must be acknowledged that these numbers do not reflect the true frequency of this event, as histopathological studies rely on selection bias of excised, suspicious lesions. In fact, the annual risk of an individual melanocytic nevus becoming malignant is extremely low and has been estimated to be approximately 0.0005% (or less than 1 in 200,000) before the age of 40 years, to 0.003% (1 in 33,000) in patients older than 60 years [2,3].

However, since many studies propose a direct correlation between the number of moles and melanoma development (roughly 2- to 14-fold), efforts to curb the rise in melanoma have centered on the detection of early changes in melanocytic nevi [2].

Observations imply that nevi undergo dynamic proliferations that appear and disappear throughout the lifetime. Currently, nevi are merged into 2 categories, congenital and acquired.

Current understanding, based on the striking, small, truly congenital nevi and nevi acquired early in life, is that the first develops before puberty, presents with a dermoscopic globular pattern, and persists for the lifetime, becoming later a dermal nevus in the adult [3,4]. In contrast, acquired melanocytic nevi develop mostly at puberty and usually undergo spontaneous involution after the fifth decade of life. However, in the realm of acquired nevi, we can distinguish 2 types of nevi: compound nevi with a superficial or with a deep dermal component. While the former is dermoscopically characterized by a reticular pattern, the latter is typified by a central elevated part showing a structureless pattern (deep dermal component) and a flat peripheral, reticular component (lateral junctional shoulders) [4,5]. Although the majority of both nevus types undergo spontaneous involution, some of the dermal components of deep compound nevi may also persist until advanced age. There is global agreement that in certain cases melanoma develops in conjunction with a preexisting melanocytic nevus.

Nevus-associated melanoma (NAM) is diagnosed on the basis of the presence of histopathological evidence of nevus components and melanoma features. Conversely, de novo melanoma (DNM) is defined as melanoma without histopathological evidence of a preexisting nevus [1]. Comparative data on melanomas that arise from preexisting melanocytic nevi and those that arise de novo is limited, and the effect of the origin of melanoma on disease characteristics and prognosis remains unclear.

Currently available data from the literature about clinical, histological, dermoscopic, and molecular features and prognosis of NAM are summarized in Table 1.

## Epidemiology

The prevalence of NAM varies across studies. Only one-third of melanomas arise in association with a preexisting nevus. The literature describes a wide range of NAM prevalence, from 4% to 72%. Lin et al reviewed 25 studies and found that 36% of melanomas were associated with a preexisting nevus [2]. Recently, Pampena et al conducted a systematic review and a meta-analysis of published reports on the NAM ratio in melanoma patients. They showed that 29.1% of melanomas developed in conjunction with a preexisting nevus and 70.9% developed de novo [1].

A possible reason for these discrepancies is that the thicker the melanoma, the higher the probability for nevus remnants to be obscured or destroyed by malignant proliferation; thus, in some cases, it is extremely difficult to determine the original association with a preexisting nevus [1,3].

## Clinical and Dermoscopic Aspects

Currently, there is widespread agreement about certain clinical features of NAM and its age- and sex-related incidence, whereas some discordance regarding the histological subtype and anatomic site is reported among different studies.

In most studies, NAM appears to be a superficial spreading type of melanoma generally occurring on the trunk. On the other hand, DNM is associated with a nodular subtype, as well as with an anatomic location on the extremities, which has better outcomes than on the trunk in several survival models [2,3].

In the meta-analysis of Pampena et al, no relevant differences were observed between NAM and DNM groups regarding the melanoma subtype and body site. Superficial spreading melanoma was the most common frequent subtype, whereas the trunk and the extremities were the most common locations [1].

To our knowledge, it is difficult to distinguish NAM and DNM based on dermoscopy, and moreover there are only a limited number of studies about this topic.

Stante et al found that an atypical pigment network and regression structures were associated with melanoma arising in a nevus [4].

To detect dermoscopic parameters, a further study by Shitara et al was conducted [5].

A case-control test set of NAM vs DNM, paired by Breslow thickness and histopathological subtype, was analyzed by 2 blinded experienced dermoscopists, according to criteria such as presence of nevus, pattern analysis, and ABCD dermoscopy score. The results showed that the presence of irregular globules, streaks, and a negative pigment network were significantly related to NAM. In contrast, the presence of a blue-white veil was not associated with NAM. No significant differences were found between the other dermoscopic criteria or in any global pattern in pattern analysis [5].

## Histopathological Features

NAM is defined by the coexistence of nevus components and melanoma features in histopathological examination. A higher prevalence of invasive melanoma is reported for both NAM and DNM groups; however, in situ melanomas are slightly more prevalent in NAMs [1].

Cymerman et al reported that DNM was associated with mean Breslow thickness greater than 1.0 mm, ulceration, and stage greater than I [3]; even Pampena et al found a significantly lower mean Breslow thickness in NAMs than in DNMs [1]. Possible reasons for these discrepancies include the possibility that nevus remnants might have been obscured by malignant cells in thicker melanoma with consequent difficulties in the measurement of the mean Breslow thickness [1,3].

The majority of NAMs are associated with acquired nevi, but, with the exclusion of congenital nevi, they are also more frequently associated with intradermal nevi or compound remnants.

Whether or not the nevus had dysplastic features does not seem to influence the prevalence of NAM. However, NAM appeared to be slightly more frequently associated with nondysplastic nevi than with dysplastic nevi [1].

## Prognosis

Certain studies suggested a more favorable overall survival with NAM, but they considered only a few factors such as age and thickness [6]. On the contrary, other authors investigated survival through Kaplan-Meier and multivariable analysis and reported no statistically significant differences in survival between patients with NAM and patients with DNM [1–3,7–9].

Lin et al found there was no correlation between NAM and sentinel lymph node status, and the presence of a nevus associated with a melanoma has no prognostic implication in overall survival [2]. Cymerman et al confirmed NAM as an independent predictor of better survival through multivariable analysis [3]. DNM is more likely to possess adverse histopathological and molecular features in primary cutaneous melanoma and appears to be an independent factor of poor outcome in multivariable analysis. Among patients with DNM, men had a statistically significant worse survival than women; there was no sex-related difference in survival among patients who were diagnosed with NAM [3].

## Genetics

In melanoma, the most common mutation is represented by BRAFV600E, which occurs in 70%–95% of patients. Kakavand et al reported that NAM had a higher frequency of BRAFV600E mutations than DNM. Indeed, 55% of NAM cases were BRAFV600E mutant and only 21% of DNM cases were BRAFV600E mutant [10].

Nevus and melanoma cells in NAMs share a similar mutational profile, which might suggest a common origin or even a malignant transformation of nevus melanocytes [3].

## Discussion

Based on daily practice, it appears that approximately 30% of melanomas are associated with a preexisting nevus, whereby it is currently impossible to predict which nevus type is at risk to undergo malignant transformation.

Tsao et al reported that the risk of an individual nevus to progress toward melanoma is higher in men aged 60+ years, who usually have few nevi [13]. However, this model does not justify the presence of melanomas in young patients with many moles.

According to our experience, current literature, and clinical evaluation, it can be assumed that there are therefore 2 different types of NAM: a melanoma that arises in the center of the mole and a melanoma that grows next to the mole. The first probably arises from a real congenital nevus (also named “congenital-like nevi,” “early-onset nevi,” or “true small congenital nevi”); the second instead comes from a compound-dysplastic acquired nevus (“late-onset nevi” or “acquired nevus”).

The “congenital-like nevi” are characterized by a globular or structureless brown pattern and, once developed, persist throughout the lifetime, acquiring finally the stereotypical shape and appearance of an intradermal nevus in the elderly. These types of nevi are mainly located in the upper region of the back and along the paravertebral area of young patients and usually are associated with a high number of nevi.

On the contrary, “acquired” nevi develop during puberty, increase in number until the fourth decade of life, and thereafter decrease via involution, apoptosis, or regression. During their development, acquired nevi undergo morphological changes that are initially characterized by a globular and later peripheral globular pattern during the active phase of growth and by a reticular or reticular mixed pattern (central structureless and peripheral reticular) during their stable phase of life.

**Figure 1:**
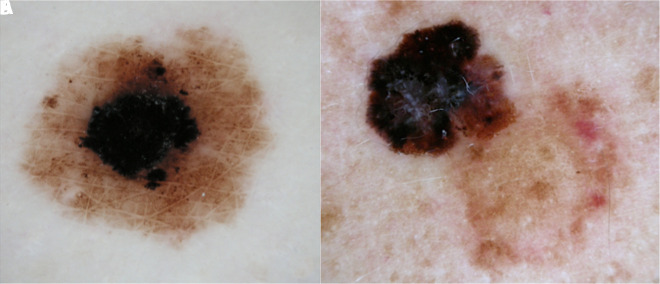
Dermoscopic features of nevus-associated melanoma (NAM). (A) NAM from congenital nevi arises in the center of the mole, and (B) NAM from acquired nevi grows next to the mole.

Dermoscopically, it has been observed that in most cases melanomas arisen from a “congenital-like” nevus come from the central portion of the lesion surrounded by a predominantly globular pattern (Figure 1A); this type of melanoma usually involves young patients (aged between 15 and 30 years) with prevalent localization on the trunk, with lower Breslow and therefore with a better prognosis.

On the other hand, the melanoma that arises on the acquired compound nevus originates from the peripheral portion of the melanocytic lesion that can be recognized through a predominantly reticular pattern (Figure 1B). The population affected by this type of NAM is typically elderly without a high number of nevi; the most typical localizations are the lower or upper limbs and the back, and the prognosis is poorer as the Breslow at diagnosis is on average more frequently elevated [11–16].

## Conclusions

According to our experience, current literature, and clinical evaluation, it can be assumed that there are therefore 2 different types of NAM: a melanoma that arises in the *center of the mole* and a melanoma that grows *next to the mole*. The first probably arises from a real congenital nevus, and the second instead comes from a compound-dysplastic acquired nevus.

Therefore, the clinical evaluation of a melanoma should consider the location and age, but also the dermoscopic pattern that allows differentiation of the cases of NAM on congenital nevus from the cases of NAM on acquired nevus. Further studies are certainly necessary to highlight possible models of progression of these melanomas according to this new concept of nevogenesis, deepening the molecular and genetic aspects and possibly correlating them to the dermoscopic characteristics.

## Figures and Tables

**Table 1:** Currently Available Data From the Literature About Clinical, Histological, Dermoscopic, and Molecular Features and Prognosis of NAM

Study	NAM/Cases	Histotype	Dermoscopy NAM	Dermoscopy DNM	Molecular Analyses NAM	Molecular Analyses DNM	Prognosis

Kaddu et al [9] 2002	148/667 (22.1%)	All	Not reported	Not reported	Not reported	Not reported	Good outcome (5-year metastasis-free survival rate 93.75%) in 69 patients with NAM

Bevona et al [11] 2003	421/1606 (26.2%)	All	Not reported	Not reported	Not reported	Not reported	Not reported

Stante et al [4] 2003	35/108 (32.4%)	All	APN: 33 (94.3%)	APN: 58 (79.4%)	Not reported	Not reported	Not reported
			IS: 26 (74.3%)	IS: 53 (72.6%)			
			IBG: 25 (71.4%)	IBG: 48 (65.7%)			
			IBD: 15 (42.8%)	IBD: 36 (49.3%)			
			BWV: (37.1%)	BWV: 38 (52%)			
			Blotches: 6 (17.1%)	Blotches: 43 (58.9%)			
			AVP: 5 (14.3%)	AVP: 27 (36.9%)			
			Regression: 21 (60%)	Regression: 29 (39.7%)			

Manganoni et al [16] 2010	10/95 (9.5%)	NM	Not reported	Not reported	Not reported	Not reported	Not reported

Betti et al [17] 2014	247/873 (28.2%)	All	Not reported	Not reported	Not reported	Not reported	DNM: MR ≥ 6 mitosis/mm^2^ can be a more important indicator of prognosis than MR < 6 mitosis/mm^2^

Shitara et al [5] 2015	390/1190 (32.7%)	All	Not reported	Not reported	Not reported	Not reported	Not reported

Kakavand et al [10] 2014	29/57 (50.8%)	All	Not reported	Not reported	BRAF V600E: 55%	BRAF V600E: 21%	Not reported

Lin et [2] 2015	235/850 (27.6%)	All	Not reported	Not reported	Not reported	Not reported	No survival difference

Haenssle et al [15] 2016	103/190 (54.2%)	All	Not reported	Not reported	>1 previous melanoma: 67 (65.1%) Multiple (>50) common and/or 3 or fewer atypical nevi: 25 (24.3%) Atypical mole syndrome: 72 (69.9%) FAMMM syndrome: 6 (5.8%)	>1 previous melanoma: 71 (81.6%) Multiple (>50) common and/or 3 or fewer atypical nevi: 12/87 (13.8%) Atypical mole syndrome: 63 (72.4%) FAMMM syndrome: 12 (13.8%)	Not reported

Cymerman et al [3] 2016	547/2149 (25.4%)	All	Not reported	Not reported	Not reported	Not reported	DNM shorter overall survival: NYU1 (HR = 1.63); NYU2 (HR = 2.52)

Alvarez Martinez et al [18] 2018	20/32 (62.5%)	All	SBW: 7 (35.0%)	SBW: 3 (25.0%)	Not reported	Not reported	Not reported
			SBBC: 3 (15.0%)	SBBC: 4 (33.3%)			
			SBBE: 0 (0.0%)	SBBE: 4 (33.3%)			
			Clods: 4 (20.0%)	Clods 4 (33.3%)			
			LR/PC: 0 (0.0%)	LR/PC: 1 (8.3%)			
			LR/PS: 2 (10.0%)	LR/PS: 0 (0.0%)			
			White lines: 8 (40.0%)	White lines: 1 (8.3%)			
			Gray/blue structures: 2 (10.0%)	Gray/blue structures: 1 (8.3%)			
			TLR/BL: 4 (20.0%)	TLR/BL: 1 (8.3%)			
			AVP: 1 (5.0%)	AVP: 2 (16.6%)			

APN = atypical pigmented network; AVP = atypical vascular pattern; BWV = blue-white veil; DNM = de novo melanoma; FAMMM syndrome = familial atypical mole and multiple melanoma syndrome; IBD = irregular black dots; IBG = irregular brown globules; IS = irregular streaks; LR/PC = lines radial or pseudopods circumferential; LR/PS = lines radial or pseudopods segmental; MR = mitotic rate; NAM = nevus-associated melanoma; NM = nodular melanoma; NYU1/2 = referring to 2 patient cohorts (see reference 3); SBBC = structureless brown-black centered; SBBE = structureless brown-black eccentric; SBW = structureless blue-white; TLR/BL = thick lines reticular or branched localized.
