# Correction

**DOI:** 10.5826/dpc.1003a99

**Published:** 2020-06-29

**Is There More Than One Road to Nevus-Associated Melanoma?**

In the Review article titled “Is there more than one road to nevus-associated melanoma?” by Vezzoni et al [1] (https://dpcj.org/index.php/dpc/article/view/dermatol-pract-concept-articleid-dp1002a28), published on April 3, 2020, the institutional affiliation of one of the authors was incorrect. Dr. Pizzichetta’s affiliations are Dermatology Clinic, Hospital Maggiore, University of Trieste and Division of Oncology B, CRO Aviano National Cancer Institute, Aviano, Italy. This article was corrected online on June 29, 2020. The editors regret the error.
